# A cross-sectional assessment of oral health status and hygiene practices of the Turkish population following the 2023 Turkiye-Syria earthquakes

**DOI:** 10.1186/s12903-025-06833-2

**Published:** 2025-09-26

**Authors:** Ezgi Sila Taskaldiran, Muhammet Bercem Saracoglu, Suleyman Emre Meseli, Cenker Zeki Koyuncuoglu

**Affiliations:** 1 Faculty of Dentistry, Department of Periodontology, Istanbul Aydin University, Istanbul, Turkey; 2 Faculty of Dentistry, Department of Periodontology, Istanbul Atlas University, Istanbul, Turkey

**Keywords:** Disaster dentistry, Oral hygiene, Postdisaster care, Orodental trauma

## Abstract

**Background:**

Natural disasters such as earthquakes disrupt not only access to basic healthcare but also routine health behaviors. Despite its relevance to overall health, oral care is often neglected in postdisaster public health responses. This study investigated oral hygiene practices and access to dental care among individuals residing in temporary shelters following the 2023 Turkiye-Syria earthquakes.

**Methods:**

A cross-sectional study was conducted with 401 participants residing in a container city in Turkiye approximately 10 weeks postdisaster. The questionnaire collected data on sociodemographic variables, pre- and postdisaster oral hygiene practices, access to oral hygiene products, and the presence of orodental trauma related to the earthquake, while routine dental screenings were conducted to assess oral health status via the Decayed-Missing-Filled Teeth (DMF-T) index and the Simplified Oral Hygiene Index (OHI-S).

**Results:**

Seventeen participants reported earthquake-related orodental trauma. While 32.4% of the participants regularly brushed their teeth before the earthquake, 92.3% reported disruption postdisaster. Toothbrush availability was 84.8%, yet interdental cleaning tool ownership remained below 2%. The mean DMFT score was 11.19 ± 8.58; the mean OHI-S score was 3.22 ± 1.40. Females and participants with toothbrush access had significantly better oral hygiene scores (*p* < 0.001). The number of remaining teeth was inversely associated with all other clinical indices (*p* < 0.001).

**Conclusion:**

The earthquake significantly disrupted oral hygiene routines and limited access to oral care tools, particularly for those who previously maintained regular habits. These findings highlight the need for postdisaster oral health support and resource distribution to mitigate long-term public health consequences and suggest that emergency relief packages should also include provisions that help maintain oral hygiene practices following disasters.

## Introduction

Earthquakes represent a category of natural disasters characterized by their capacity to endanger human life and inflict significant damage. The overturning of objects, the sudden movement of individuals and the collapse of buildings during seismic activity have the potential to cause physical problems. Additionally, psychological issues may manifest in individuals depending on whether they have experienced or witnessed an earthquake [1]. Following earthquakes, alterations in the living circumstances of victims are a predictable consequence [2]. Research indicates that the psychological difficulties of individuals can persist for up to two years following the Great Japan Earthquake of 2011 [3].

On 6 February 2023, two earthquakes, with magnitudes of 7.8 and 7.6 on the Richter scale, occurred approximately nine hours apart in southeastern Turkiye. The earthquakes affected 11 major provinces in Turkiye and neighboring Syria. The area affected included a region traversed by active fault lines, and these faults have the potential to produce earthquakes with a significant magnitude of impact. Despite the high level of earthquake awareness in Turkiye, the situation has reached a catastrophic dimension due to a confluence of factors, including housing instability, challenging seasonal conditions, and the size of the affected area [4]. A report published by the Republic of Turkiye Ministry of Environment, Urbanization and Climate Change in the aftermath of the earthquake revealed that approximately 156,000 buildings had been destroyed or severely damaged, with an additional 821,302 buildings requiring reconstruction [5]. The issue of homelessness resulting from destruction has been addressed through the construction of temporary housing solutions, including the erection of tent cities and container communities [6]. In addition to alterations in living conditions and physical and emotional trauma directly associated with earthquakes, the potential for the emergence of epidemics in the region has emerged as a significant concern [7].

The role of dentists has often been studied after deadly earthquakes and other natural disasters in different parts of the world. The most common focus has been the role of dentists in identifying disaster victims [8–10]. Furthermore, studies have been conducted to ascertain the dental status of individuals following the occurrence of natural disasters. The practice of oral hygiene becomes more challenging, and the incidence of caries increases in individuals affected by earthquakes [11]. In the postdisaster period following the Great East Japan Earthquake and Tsunami, the oral health-related quality of life of victims was reported to be low because of the loss of teeth and dentures [12]. Furthermore, in a different study conducted among Nepalese individuals residing in provisional accommodations, dental plaque and calculus were present in more than 90% of the subjects, and high levels of gingival inflammation were evident [13].

Determining the status of individuals who have oral hygiene tools after large-scale disasters and taking the necessary precautions in this direction are highly important. To the best of our knowledge, no similar study has been conducted in this area. Consequently, the objective of this study is twofold: first, to ascertain the oral and dental health status of individuals affected by the 6 February 2023 Turkiye-Syria earthquakes and residing in temporary shelters; second, to draw inferences about potential oral and dental health needs that may arise in the aftermath of the earthquakes.

## Methods

### Study design

The study was approved by the Istanbul Aydin University Non-Interventional Clinical Applications Ethics Committee on 4 October 2023, with the approval number 2023/112 and was performed in accordance with the principles of the Declaration of Helsinki. Informed consent has been obtained from all the participants to participate in this study.

### Study population

The dataset comprises the findings of routine dental examinations recorded in the Istanbul Aydın University Education and Living Complex, which is located in Gaziantep Nurdagi Container City, the location where disaster victims affected by earthquakes on 6 February 2023 resided. The findings were derived from data collected approximately 10 weeks after the earthquakes (25/04/2023-09/05/2023) by a volunteer dentist (MBS) affiliated with the Faculty of Dentistry of Istanbul Aydin University, who was engaged in relief activities in the container city. All clinical examinations were performed by this examiner. Considering the recommendations described in the WHO Oral Health Surveys: Basic Methods [14], 10 individuals who were not included in the main study sample were examined twice at 3-hour intervals by the designated examiner, focusing on the debris score component of the OHI-S index [15]. Intra-examiner reliability was assessed by comparing these repeated observations. The Cohen’s Kappa coefficient was calculated as 0.85, indicating “almost perfect” agreement [16]. All participants provided consent for the scientific use of the data obtained.

The first evaluation output of the study was chosen as the plaque index showing the oral care level of the disaster victims. On the basis of the reference values of Rokaya et al. [13], the smallest sample size that could reveal a real difference of 0.5 units in the plaque index with an effect size of 0.16, 95% power and 0.05 margin of error was found to be *n* = 425 with the one sample test. The inclusion criteria were being aged between 18 and 80 years, having experienced the Turkiye-Syria earthquakes, currently residing in Gaziantep Nurdagi Container City and providing informed consent for participation and the use of data for scientific purposes. Individuals who were not affected by the earthquake and those with fewer than five teeth in the oral cavity were excluded from the study because of limitations in assessing periodontal and dental health.

### Data collection tool and study protocol

The data collection tool, which was specifically designed for the routine dental screening of disaster victims, consisted of two sections.

The first section included demographic questions about the participants’ age and sex, as well as six yes/no questions to assess oral care habits, for a total of eight questions. These questions assessed regular tooth brushing habits, the impact of the earthquake on these habits, and the availability of oral hygiene materials (toothbrush, toothpaste, dental floss, interdental brushes). Individuals who reported brushing their teeth at least twice a day prior to the earthquake were classified as regular brushers. Additionally, participants were asked whether they had experienced any orodental trauma, which is defined as any acute injury to the teeth, supporting periodontal tissues, oral soft tissues (such as lips, cheeks, or tongue), or maxillofacial bones caused by external physical forces [17].

The second section involved the assessment of decayed, missing and filled teeth via the Decayed-Missing-Filled Teeth (DMF-T) index and the Simplified Oral Hygiene Index (OHI-S) defined by Greene and Vermillion [15]. The DMF-T index was calculated in accordance with the guidelines set out in the WHO Oral Health Surveys Basic Methods document. The number of decayed (D), missing (M) and filled (F) permanent teeth was summed to yield a total DMF-T score [14]. The OHI-S assesses oral hygiene by measuring the amount of dental biofilm (debris score) for short-term hygiene assessment and the amount of dental calculus (calculus score) for long-term hygiene assessment. The scores were taken from teeth 16, 11, 26, 36, 31 and 46. If these teeth were missing, the scores were taken from teeth 17–18, 21, 27–28, 37–38, 41 and 47–48. The debris and calculus scores were each scored between 0 and 3, and their sum was used to calculate the OHI-S score.

### Statistical analysis

The statistical analyses of the data used in the study were performed via the Statistical Package PASW 20.0 (SPSS, Chicago, IL, USA). Descriptive statistics are presented as frequencies, percentages, means, and standard deviations. The Mann‒Whitney U test was used to compare the means of variables between sex groups. Spearman’s correlation analysis was employed to evaluate relationships between variables. Statistical significance was considered at a level of *p* < 0.05.

## Results

A total of 468 participants were initially included in the study. However, 34 were excluded because they were outside the age range of 18–80 and 33 were excluded because they had fewer than five teeth. As a result, 401 individuals were included in the final analysis. Among the 401 participants, 269 were female and 132 were male. The mean age of the participants was 42.75 years (female: 42.36 years; male: 43.52 years).

Seventeen individuals had a history of traumatic injury to the head and neck region as a result of the earthquake. Among these individuals, four experienced tooth loss, and one experienced a jaw fracture. Additionally, one participant reported the loss of a removable prosthesis during the earthquake.

The availability of essential oral hygiene products, including toothbrushes, toothpaste, dental floss, and interdental brushes, is presented in Table 1. Prior to the earthquake, 32.4% (*n* = 130) of the participants reported brushing their teeth regularly, whereas 66.6% (*n* = 271) did not maintain a regular tooth brushing routine. Following the disaster, 92.3% (*n* = 120) of those who previously brushed regularly and 22.8% (*n* = 48) of those who did not report that their brushing habits were further disrupted.

**Table 1:** Availability of oral hygiene materials

	Availability (%)	Frequency (*n*)
Toothbrush	84.8%	340
Toothpaste	82.2%	330
Dental Floss	1.49%	6
Interdental Brush	0.99%	4

The mean clinical index scores of all participants in the study were determined to be 1.75 ± 0.70 for the debris index, 1.47 ± 0.75 for the calculus index, 3.22 ± 1.40 for the OHI-S, and 11.19 ± 8.58 for the DMFT; the mean number of remaining teeth was determined to be 23.26 ± 5.68. The mean debris, calculus, and OHI-S scores for females were significantly lower than those for males (for all: *p* < 0.001), as presented in Table 2. Additionally, participants with a toothbrush presented significantly lower debris, calculus, and OHI-S scores than did those without a toothbrush (*p* = 0.002, *p* = 0.001, and *p* = 0.001, respectively).

**Table 2:** Comparison of oral hygiene indices and DMF-T by gender

	Female (*n* = 269)	Male (*n* = 132)	*p*
Debris Index	1.65 ± 0.66	1.95 ± 0.73	< 0.001
Calculus Index	1.36 ± 0.67	1.69 ± 0.85	< 0.001
OHI-S	3.02 ± 1.27	3.65 ± 1.53	< 0.001
DMF-T	11.55 ± 8.65	10.48 ± 8.99	0.061
Remaining teeth	23.14 ± 5.54	23.50 ± 5.96	0.184

The data are presented as the means ± standard deviations

*Abbreviations*: *OHI-S* Simplified Oral Hygiene Index, *DMFT* Decayed, Missing, Filled Teeth Index

The relationships between the variables examined are shown in Table 3. The number of remaining teeth was found to have a significant inverse relationship with all the variables examined, whereas all the other variables were found to have a significant positive relationship (all *p* < 0.001).

**Table 3:** Correlations of oral hygiene indicators and age

		Debris Index	Calculus Index	OHI-S	DMFT	Remaining teeth	Age
Debris Index	r	1	0.813	0.949	0.230	−0.317	0.304
Calculus Index	r	0.813	1	0.951	0.268	−0.338	0.360
OHI-S	r	0.949	0.951	1	0.261	−0.347	0.352
DMFT	r	0.230	0.268	0.261	1	−0.759	0.576
Remaining teeth	r	−0.317	−0.338	−0.347	−0.759	1	−0.616
Age	r	0.304	0.360	0.352	0.576	−0.616	1

The Spearman correlation coefficient (r) for all the data were significant at the *p* < 0.001 level

## Discussion

The two successive major earthquakes that struck Turkiye in 2023 had a significant impact on nearly 15 million people and caused extensive destruction across numerous provinces and districts. Official reports indicate that the death toll resulting from these earthquakes exceeds 50,000 [18]. Earthquakes represent one of the most destructive seismic events recorded in the region’s recent history. Occurring during winter months marked by harsh cold and heavy snowfall, the disaster was further exacerbated, as these conditions significantly hindered relief and rescue efforts, increasing the severity of the catastrophe [19]. The Disaster and Emergency Management Presidency of Turkiye has declared a “Level 4” state of emergency, the highest level outlined in the Disaster Response Plan of Turkiye, signifying the need for international assistance. Similarly, the World Health Organization issued a “Level 3” alert in response to earthquakes that struck Turkiye. In response to the appeals for assistance, 11,320 search and rescue personnel from 90 different countries were deployed to Turkiye to provide support for relief operations [20, 21].

Challenges in meeting the clean water needs of earthquake survivors, along with food shortages and housing inadequacies, have forced individuals to adapt to altered living conditions. Moreover, difficulties in accessing medications and damage to healthcare facilities, including hospitals, have significantly diminished the capacity for emergency medical care. Large-scale population displacement, restricted access to clean water sources, and challenges in the removal of deceased individuals from residential areas have collectively heightened the risk of infectious diseases in the postearthquake period [22, 23]. In addition to the risk of infections, earthquakes frequently result in trauma to the extremities as well as injuries to the head and neck regions [24].

While the current study focuses on the 2023 Turkiye-Syria earthquakes, previous large-scale seismic disasters, such as the 9.5 magnitude 1960 Valdivia earthquake in Chile and the 1976 Tangshan earthquake in China, which caused the deaths of more than 250,000 people, provide important historical lessons in terms of the fragility of health systems and emergency preparedness. Although oral and dental health was not systematically documented during these previous events, the absence of integrated dental health intervention strategies and inadequate access to hygiene tools and health infrastructure clearly exacerbated public health outcomes in the aftermath of the disasters. The lessons learned from these events emphasise the need to include oral and dental health provisions in future disaster risk reduction plans [25].

In the literature, earthquake dentistry has been confined predominantly to identification studies, with existing research often limited by data constraints. The present study aims to address this gap by assessing the dental status, availability of oral care materials, and oral hygiene practices among individuals affected by the Turkiye–Syria earthquakes.

Due to the inadequacy of field conditions in terms of providing additional materials and the limited time available for examining and evaluating each participant during the study, the OHI-S and DMF-T indices, which are frequently used in epidemiological studies, were selected.

The findings of this study demonstrate a notable lack of adequate hygiene materials, particularly interdental cleaning tools, within temporary living accommodations established in the aftermath of the earthquake. Prior to the disaster, national studies conducted in Turkiye [26, 27] reported toothbrush ownership rates ranging from 84 to 95%. Similarly, the data demonstrate that the prevalence of toothbrush ownership in earthquake-affected regions falls within the aforementioned range. However, the availability of interdental cleaning tools, including dental floss and interdental brushes, is markedly lower in these areas than the national average. A previous study revealed that 15% of the population owned dental floss, whereas only 4% owned interdental brushes [27]. These findings demonstrate that, while interdental cleaning tool ownership was already limited at the national level, the earthquake further exacerbated this deficiency, reducing availability to critical levels in affected regions.

The maintenance of oral hygiene practices after major disasters plays a crucial role in preventing difficult-to-treat infectious diseases such as pneumonia, especially in people with a predisposition to infection [28]. However, difficulties in accessing water frequently result in disruptions to oral hygiene routines. A recent study demonstrated a decline in the frequency of toothbrushing among children following earthquakes [29]. Similarly, our study indicates that almost all individuals with previously established oral hygiene routines reported disruptions due to the earthquake, with more pronounced effects observed in adults. These findings emphasize the susceptibility of oral hygiene behaviors to external stressors, particularly in the context of disasters. Furthermore, the displacement from home and the subsequent adaptation to a new living environment, coupled with the physical and psychological trauma experienced, may result in survivors being unable to maintain their previous hygiene routines at the same level [23, 24].

Our findings indicate that both male and female participants presented higher debris index scores than calculus index scores did. This observation suggests that earthquake victims are currently facing challenges in maintaining their daily hygiene routines, as the debris index reflects short-term plaque accumulation, whereas the calculus index represents longer-term deposits. Furthermore, compared with female participants, male participants were found to have significantly greater debris, calculus, and OHI-S scores. This finding supports the notion that men generally exhibit weaker oral hygiene practices than women do, which is consistent with previously reported trends in the literature [30]. In addition, the high DMF-T index observed among all participants highlights the urgent need for dental care following the disaster.

A review identified head injuries as the third most common type of trauma associated with earthquakes [24]. On the basis of this information, injuries to the dental and oral regions might also be frequently observed. However, in our study, a history of orodental trauma was recorded in only 17 individuals. This finding may be attributed to the high mortality rates associated with earthquake-related head and neck injuries, as well as the likelihood that individuals with injuries in these areas require specialized care.

Although the present study aimed to determine the dental status of survivors after an earthquake, similar studies conducted following other natural disasters were also reviewed. To exemplify this, a recent systematic review underscored the absence of a comprehensive protocol that unequivocally delineates the role of dentists, particularly within vulnerable demographics such as children, in the aftermath of disasters [31]. A further review concluded that, while there is a possibility that earthquakes may have a detrimental effect on oral health, there is an absence of sufficient evidence to demonstrate a correlation between other natural disasters and oral health [32]. Furthermore, Shiba et al. found that oral health declined in the long term after the disaster, particularly among older individuals, along with psychological well-being. They also found that individuals who lost their homes had difficulty maintaining their daily habits [33]. Conversely, the present study demonstrated that post-earthquake relocation significantly impacts dental hygiene practices. These results suggest that, in the aftermath of any kind of disaster, oral health is a neglected area of care.

Although the results of this study provide important data on the periodontal and dental status and oral hygiene habits of earthquake victims, it also has several limitations. As the study sample was limited to individuals residing in a single container city, the generalizability to other affected regions remains limited. In addition, because of the ongoing intense emotional impact of the earthquake, important variables such as the psychosocial status and quality of life of earthquake victims could not be assessed. Finally, although the OHI-S and DMF-T indices were recorded via intraoral examination, more detailed examination methods, such as dental radiographs, could not be used because of the inappropriate conditions in the region.

## Conclusion

The findings from this study underline the critical need for oral health interventions in disaster-prone areas, emphasizing the integration of dental hygiene supply chains into emergency response frameworks. This suggests that the difficulties associated with the provision of health services and hygiene supplies in the aftermath of the disaster may have contributed to the exacerbation of these issues. Therefore, future research should focus on developing effective strategies to support the maintenance of oral hygiene in postdisaster settings. Additionally, the implementation of awareness and educational initiatives aimed at improving oral hygiene practices, particularly in disaster-affected regions, is strongly recommended.

## Data Availability

No datasets were generated or analysed during the current study.
